# Electrical impedance tomography in anaesthetised chickens (*Gallus domesticus*)

**DOI:** 10.3389/fvets.2024.1202931

**Published:** 2024-03-13

**Authors:** Adrian M. Wong, Hei Y. Lum, Gabrielle C. Musk, Timothy H. Hyndman, Andreas D. Waldmann, Deborah J. Monks, Ross S. Bowden, Martina Mosing

**Affiliations:** ^1^School of Veterinary Medicine, Murdoch University, Perth, WA, Australia; ^2^Animal Care Services, University of Western Australia, Perth, WA, Australia; ^3^Department of Anaesthesiology and Intensive Care Medicine, Rostock University Medical Centre, Rostock, Germany; ^4^Brisbane Bird and Exotics Veterinary Service, Brisbane, QLD, Australia; ^5^Anaesthesiology and Perioperative Intensive Care, University of Veterinary Medicine, Vienna, Austria

**Keywords:** air sacs, avian, birds, breathing pattern, distribution of ventilation, recumbency, ventilation

## Abstract

The applicability of electrical impedance tomography (EIT) in birds is unknown. This study aimed to evaluate the use of EIT in anaesthetised chickens in four recumbency positions. Four adult Hyline chickens were anaesthetised with isoflurane in oxygen, and intubated endotracheally for computed tomography (CT). A rubber belt was placed around the coelom caudal to the shoulder joint. A chicken-specific finite element (FE) model, which is essential to generate anatomically accurate functional EIT images for analysis, was constructed based on the CT images obtained at the belt level. Ten additional chickens were anaesthetised with the same protocol. An EIT electrode belt was placed at the same location. The chickens were breathing spontaneously and positioned in dorsal, ventral, right and left lateral recumbency in a randomised order. For each recumbency, raw EIT data were collected over 2 min after 13 min of stabilisation. The data were reconstructed into functional EIT images. EIT variables including tidal impedance variation (TIV), centre of ventilation right to left (CoV_RL_) and ventral to dorsal (CoV_VD_), right to left (RL) ratio, impedance change (ΔZ) and eight regional impedance changes including the dorsal, central-dorsal, central-ventral and ventral regions of the right and left regions were analysed. Four breathing patterns (BrP) were observed and categorised based on the expiratory curve. A linear mixed model was used to compare EIT variables between recumbencies. Fisher's exact test was used to compare the frequencies of breathing patterns for each recumbency. The ΔZ observed was synchronous to ventilation, and represented tidal volume of the cranial air sacs as confirmed by CT. Significant differences were found in CoV_VD_ and regional impedance changes between dorsal and ventral recumbencies (*P* < 0.05), and in CoV_RL_, RL ratio and regional impedance changes between right and left recumbencies (*P* < 0.05), which suggested a tendency for the distribution of ventilation to shift towards non-dependent air sacs. No differences were found for TIV and respiratory rate between recumbencies. Recumbency had a significant effect on the frequencies of each of the four BrPs (*P* = 0.001). EIT can monitor the magnitude and distribution of ventilation of the cranial air sacs in different recumbencies in anaesthetised chickens.

## 1 Introduction

General anaesthesia is an essential aspect of avian medicine and is routinely used for diagnostic purposes due to their non-compliant nature, and for surgical procedures to produce unconsciousness, muscle relaxation and analgesia to maintain patient welfare against nociceptive stimulations (1). General anaesthesia is however associated with higher mortality rates (2, 3) than what has been reported in dogs, cats, rabbits and horses (4, 5).

Depression of the respiratory system during general anaesthesia of birds has been proposed as a risk factor for anaesthesia-related morbidity and mortality, as they have high metabolic requirements that require efficient gas exchange (6). Alterations to blood gases such as hypercapnia and hypoxaemia can lead to acid-base derangements and tissue hypoxia, respectively. Peri-anaesthetic monitoring of the respiratory system is therefore crucial to allow recognition of complications and timely interventions to minimise patient morbidity and mortality (7). In mammals, monitoring of the respiratory system during anaesthesia is usually achieved with the use of capnography, pulse oximetry, blood gas analysis and occasionally spirometry. However, accuracy of capnography has been shown to be variable in birds (8), pulse oximeters are not calibrated for birds (1) and spirometry is rarely used in a clinical setting. Techniques to evaluate the avian respiratory system accurately is lacking.

Electrical impedance tomography (EIT) is a non-invasive real-time imaging modality that has gained considerable interest in veterinary medicine for respiratory monitoring over the last decade (9). A belt with 16 to 32 equidistant electrodes is placed around the thorax and a non-perceivable alternating current (AC) is applied to the body through pairs of electrodes with the voltage measured by the rest. As air and tissue have different conductivity, the impedance of the thorax changes throughout the respiratory cycle. The signals obtained by EIT are termed impedance change (ΔZ) and are measured in arbitrary units (AU).

The EIT data obtained by the boundary voltage measurements can be reconstructed into functional images, using a species-specific finite element (FE) model (10, 11). A FE model is constructed based on a transverse image of the thorax levelled at the proposed belt position, typically by computed tomography (CT). Within the tomographic image, the outlines of specific organs such as the lungs and cardiac silhouette can be digitally segmented as regions of interest (ROI). The contours of these ROIs are then used to generate the FE model. By mapping the precise thoracic anatomy of the species of interest, anatomically accurate EIT images can be generated (12). With these functional EIT images, the distribution of ventilation within the lungs can be quantified on a breath-to-breath basis in real time (13–16); a feature distinct to other respiratory monitoring technologies. Additionally, EIT can estimate tidal volume based on its linear relationship with ΔZ (17–20), also termed tidal impedance variation (TIV) (21). Further comprehensive information on the technical background and veterinary application of EIT can be referred to the thoracic EIT veterinary consensus statement (9).

**Figure 1:**
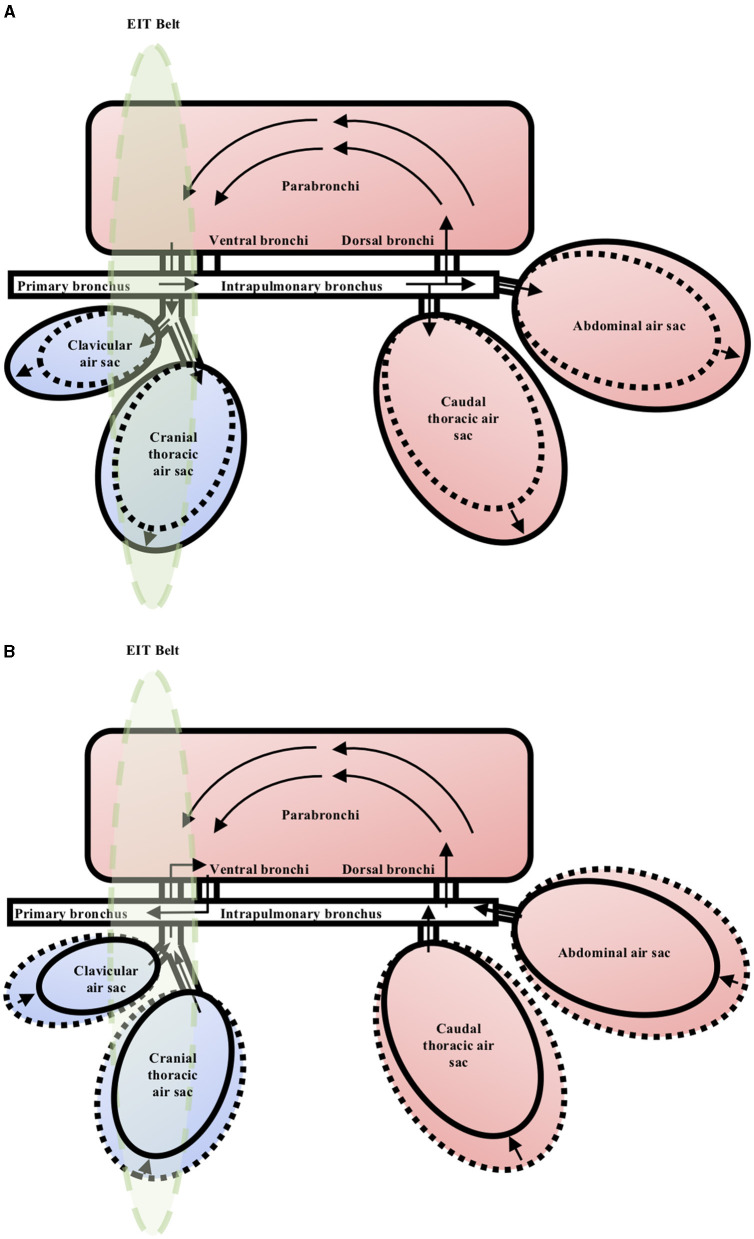
Schematic representation of the respiratory system in the chicken during the respiratory cycle. The arrows indicate direction of gas flow. The green ellipse represents the area of coelom which data will be captured by the EIT belt. **(A)** Inspiration. The air sacs expand and get filled by tidal gas during inspiration. The negative pressure within the expanded cranial air sacs drives gas flow through the parabronchi unidirectionally. **(B)** Expiration. The air sacs contract and empty the tidal gas during expiration. The positive pressure within the caudal air sacs drives gas flow through the parabronchi unidirectionally.

There are substantial differences between the avian and mammalian respiratory systems. The primary difference is that in birds, there is a functional separation of ventilation and gas exchange. The compliable air sacs are not involved in gas exchange. They are responsible for generating tidal flow that maintains unidirectional air flow through the parabronchi (tertiary bronchi) at both inspiration and expiration. From the parabronchi, air moves through atria, infundibula and into air capillaries, where the majority of gas exchange occurs (6, 22). When referring to the avian lung, it is to be referred to the gas exchange component of the avian respiratory system, which does not include the air sacs. During inspiration, the negative pressure within the expanded cranial air sacs drives air flow through the non-compliable parabronchi, and during expiration, the positive pressure within the caudal air sacs again drives air flow unidirectionally through the parabronchi (6, 22) (Figure 1). This arrangement allows a much more efficient extraction of oxygen when compared to mammals, sustaining their innately high metabolic rate (1, 23, 24).

It has been proposed that ΔZ in the mammalian lung represents the stretch and deformation of epithelial cells, blood, capillaries and extra-capillary blood vessels secondary to alveolar expansion, instead of the volume of gas gained during inspiration (25, 26). Conversely in birds, the air sacs are only thinly lined with simple squamous epithelial cells and contain minimal blood vessels (27). It is therefore uncertain whether ΔZ is present in the avian air sacs.

Given the anatomical and physiological differences in the respiratory system of mammals and birds, it is unknown if EIT can be used to monitor the respiratory system of birds. This study will be the first to document the application of EIT in this class of vertebrates.

The primary aim of this study was to evaluate the feasibility of EIT to monitor ventilation in chickens. The first objective was to verify that the ΔZ can be measured and is synchronous to breathing. The second objective was to identify the anatomic structures that generate the ΔZ. The third objective was to evaluate its ability to detect differences in EIT variables when challenged by a change in recumbency. It was hypothesised that EIT can measure ΔZ in synchronicity with ventilation in the air sacs but not the lungs, and that EIT can detect differences of EIT variables when chickens are placed into different recumbencies. The secondary aim of the study was to report the EIT variables in the four recumbencies. It was hypothesised that recumbencies will significantly affect EIT variables.

## 2 Materials and Methods

### 2.1 Ethical approval

This study was approved by the animal ethics committee of Murdoch University (permit number R3074/18).

### 2.2 Animals

Fourteen, 14-month-old adult female Hyline chickens were included in this study. The mean (±SD) body weight was 1.89 kg ± 0.36 kg.

Sample size calculation was not possible as no prior data were available, due to the novelty of the topic in birds. Furthermore, the recruited population was a convenience sample. These chickens were initially involved in an unrelated observational study at the same institute and were included in this study after finishing the initial study but before euthanasia. The chickens were housed in a dedicated holding facility, with access to food and water until the evening prior to the study. The general wellbeing of the chickens was assessed and confirmed by a veterinarian once daily based on general demeanour, appearance (e.g., gait, feather and skin condition), food intake, and egg and faecal production.

### 2.3 Anaesthesia

Each chicken was restrained in a towel and positioned in sternal recumbency. Anaesthesia was induced with isoflurane (vaporiser setting of 5%) (IsoFlo, Zoetis, Australia) in oxygen administered through a face mask. Fresh gas flow with 100% oxygen was set to 3 L/min. Orotracheal intubation was then performed using a cuffed 3.5 mm internal diameter endotracheal tube, once the chickens were adequately anaesthetised. Cuffed endotracheal tubes were used in this study to avoid leakage of gas and therefore to facilitate an inspiratory hold for CT. It should be noted that uncuffed tubes are typically recommended for birds due to their complete tracheal rings, and the associated risk of mucosal and tracheal damage (6). The endotracheal tube was attached to a paediatric breathing system for the chickens that underwent CT, and to a Mapleson D non-rebreathing system (Bain system) for the chickens that underwent the study protocol. Anaesthesia was maintained with isoflurane (vaporizer setting of 2%) in 100% oxygen with a fresh gas flow rate of 1-2 L/min. The chickens breathed spontaneously. Standard physiological variables including heart rate (HR), respiratory rate (RR), side-stream end-tidal carbon dioxide (ETCO_2_), peripheral haemoglobin saturation of oxygen (S_P_O_2_), and cloacal temperature (T), were continuously monitored by a dedicated coinvestigator during anaesthesia using a multiparameter monitor (SurgiVet Advisor Vital Signs Multi-parameter Monitor, Smiths Medical, NSW, Australia).

At the end of the study period, each anaesthetised chicken was euthanised with pentobarbitol 160 mg/kg (Valabarb, Jurox, NSW, Australia) administered directly into the brachial vein. Asystole was confirmed by auscultation.

### 2.4 CT and FE model construction

Four of the fourteen chickens underwent CT (Aquilion Lightning 80, Canon Medical Systems) of the coelom in dorsal recumbency. A sham EIT belt consisting of a rubber tube was placed 5 cm caudal to the thoracic inlet, at the cranial point of the sternum, behind the shoulder joint and beneath the wings.

The CT images (Settings: 0.5 mm slice, 120 kV, dynamic mA, pitch 0.813) were obtained during the end-inspiratory and end-expiratory period. End-inspiratory images were obtained during inspiratory hold, by manually closing the adjustable pressure-limiting valve and gently squeezing the rebreathing bag to a positive pressure of 5 cmH_2_O. Following inspiratory hold, the chickens would consistently enter a temporary period of apnoea, which facilitated the end-expiratory images to be obtained. After the CT, the chickens were euthanised as described.

**Figure 2:**
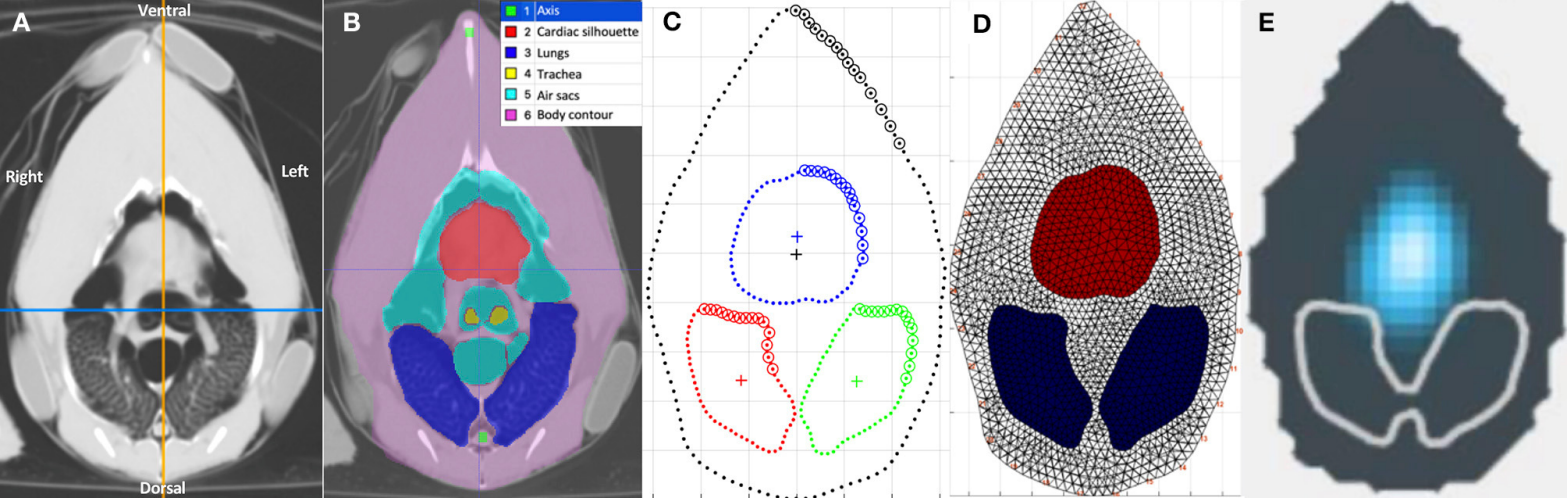
Tomographic images of the coelom at the level of the EIT belt, in various formats. The images are in sequential order from left to right, **(A–E)** detailing the process of generating a chicken-specific functional EIT image. All images are aligned in the same orientation as labeled in **(A)**. **(A)** Computed tomographic (CT) image of the coelom at the level of the EIT belt. **(B)** Segmentation of the regions of interest (ROI), including the cardiac silhouette, air sacs, lungs, tracheal bifurcation and the body contour, using the software ITK-SNAP. Each of the segmented structures are shaded with its respective colour as indicated in the legend. **(C)** Model of the coelom using the mean contours of the segmentations from the CT images. The blue outlines the cardiac silhouette, the red outlines the right lung, and the green outlines the left lung. **(D)** The chicken-specific finite element (FE) model, created using the software Matlab. The red area represents the cardiac silhouette, and the blue represents the lungs. **(E)** Functional EIT image, after application of the FE model on the raw EIT data, during inspiration in one chicken. The blue represents impedance change, the gray outlines the outer contour, and the white outlines the lungs ROI. No impedance change was noted within the lungs ROI. Impedance signals were noted in the area of the air sacs and cardiac silhouette.

**Figure 3:**
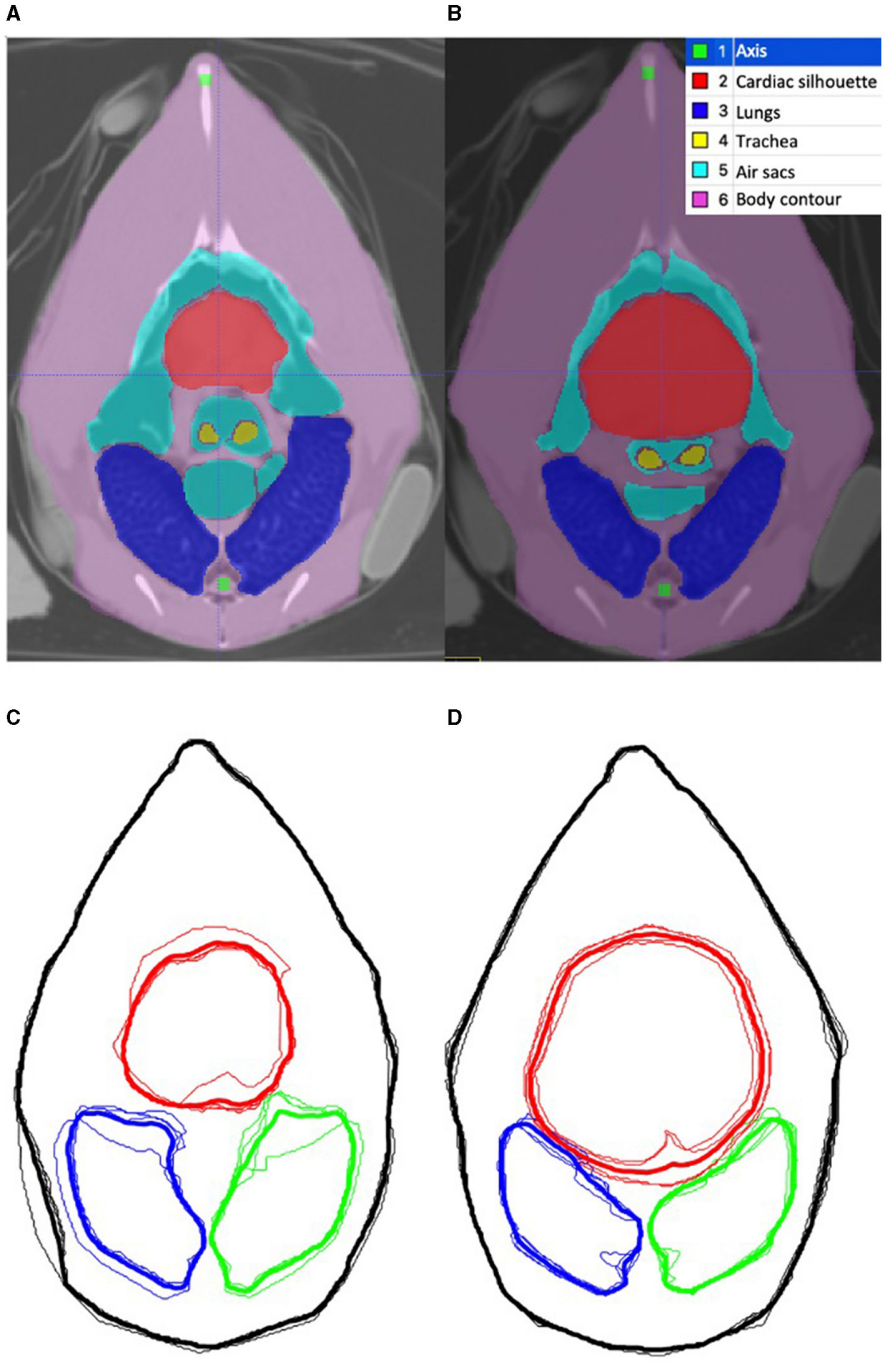
Tomographic images of the coelom at the level of the belt during inspiration and expiration. **(A, B)** are computed tomographic images of the coelom in one chicken during inspiration and expiration, respectively. The segmented region of interests (ROI) are labeled in the legend. **(C, D)** are the corresponding outline contour of the ROI based on the four chickens that underwent CT during inspiration and expiration, respectively. The thin line represents each individual chicken, and the thick line represents the mean contour. The red outlines the cardiac silhouette, the blue outlines the right lung and the green outlines the left lung. The body contour, skeletal structures remained static in size. The area of the air sacs increases during inspiration and decreases during expiration. The area of the cardiac silhouette increases during expiration and decreases during inspiration.

For each chicken, the end-inspiratory tomographic image at the level of the sham belt was used as a reference for the initial ROI segmentation (Figure 2A). The outer body contour, lungs, cardiac silhouette, air sacs, trachea bifurcation and oesophagus were identified as ROIs using a dedicated software (ITK-SNAP) (28) (Figure 2B). Based on the technique described in mammals, the chicken-specific finite element (FE) model was constructed using the mean contours of the outer contour, lungs and cardiac silhouette as ROIs from the four chickens (Figures 2C, D), using a programming software (Matlab 2017b, Mathworks) (29, 30). Adaptation to include the cranial air sacs into the model was deemed not possible based on the complex superimposition of the air sacs and the cardiac silhouette between inspiration and expiration (Figures 3A, B).

As per convention, the mean contours of the outer body contour, cardiac silhouette, and lungs from the four chickens were then calculated and aligned to construct the corresponding mean FE model using a programming software (Matlab 2017b, Mathworks) (Figure 2D).

Additionally, the movement and changes of the organs within the coelom during the respiratory cycle were evaluated by CT imaging. The segmentation process as described was repeated for the expiratory tomographic images of the coelom. The mean contours during inspiration and the mean contours during expiration were graphically superimposed, allowing visual comparison of the changes to the ROI contours within the coelom during the respiratory cycle. The changes were descriptively analysed.

### 2.5 Data collection

**Figure 4:**
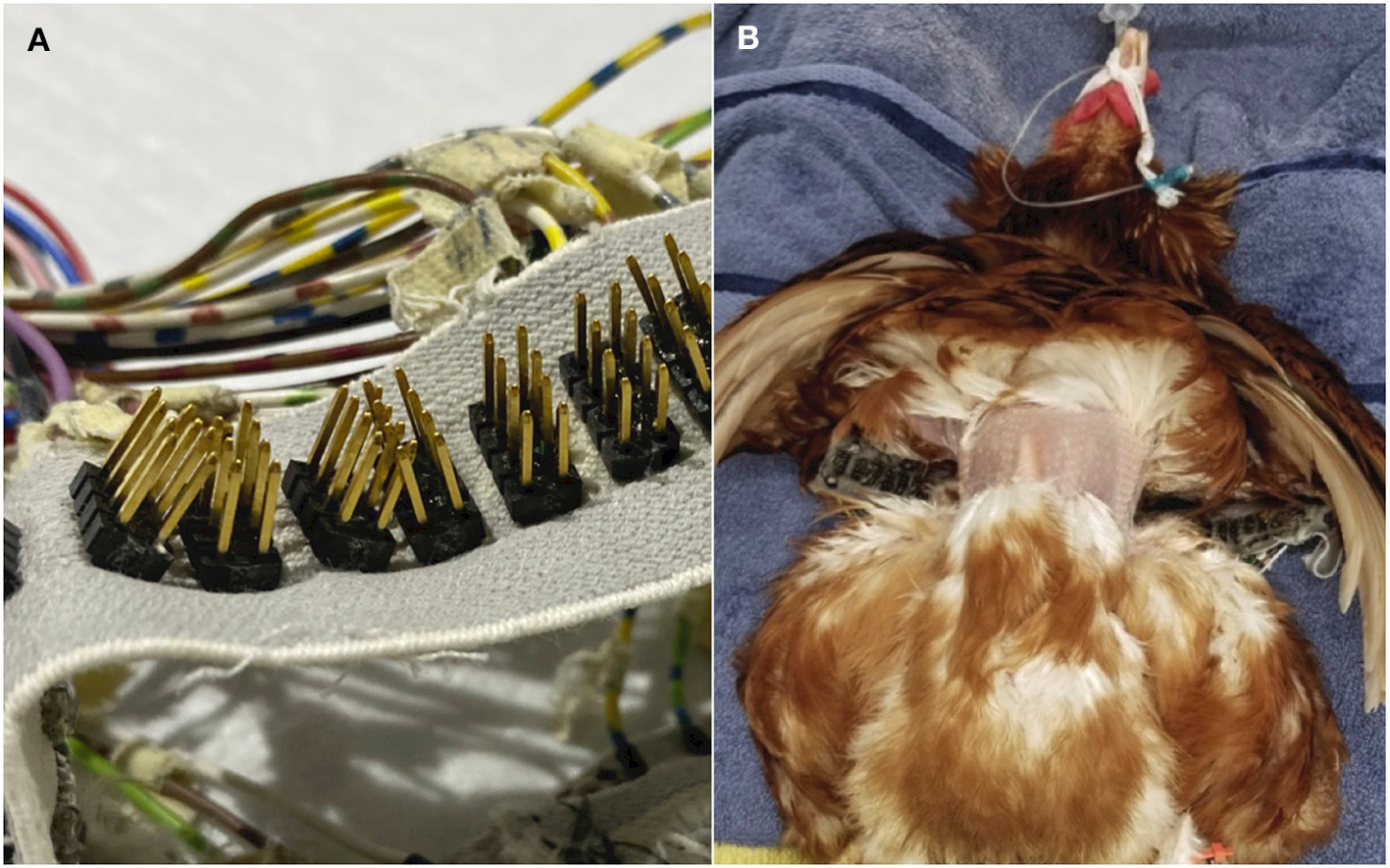
**(A)** The EIT belt utilised in the study, with 32 equidistant electrodes fitted in an elastic belt. Each electrode consisted of 12 gold plated pin arrays. **(B)** Photographic image of one chicken in the experimental protocol, positioned in dorsal recumbency, with a circumferential band of feathers removed and the EIT belt positioned just caudal to the shoulders.

Ten of the fourteen recruited chickens underwent the study protocol with the EIT electrode belt. The EIT belt was constructed for a previous study in lambs (31). The belt included 32 equidistantly placed electrodes on an elastic woven band (Figure 4A). Each electrode had 12 gold plated pin arrays to allow adequate skin contact (Figure 4B).

A circumferential band of feathers ~4 cm wide was first removed from the thorax caudal to the shoulder joint, at the level of the cranial sternum (Figure 4B). Low-conductive ultrasound gel was generously applied onto each electrode and onto the skin of the thorax before the belt was positioned around the thorax. The belt was placed 5 cm caudal to the thoracic inlet, at the cranial point of the sternum, behind the shoulder joints and beneath the wings. A stay suture was placed at the cranial point of the sternum to prevent the belt from slipping during recumbency changes. The belt was manually checked to be of suitable tightness around the coelom to minimise interference to breathing, by observing adequate coelom inflation and deflation during tidal ventilation. Sufficient skin contact between the belt and the coelom was also ensured, using the inbuilt function of the EIT software that highlights adequate electrode contact and contact failure (BB Vet SW, SenTec, Switzerland).

The chickens were placed in all four recumbency positions (dorsal, ventral, right lateral and left lateral) in a randomised order. The chickens remained in each recumbency position for 15 min: 13 min of stabilisation followed by 2 min of EIT data recording using the EIT software (BB Vet SW, SenTec, Switzerland). The EIT system applies an alternating current of 192 kHz at an amplitude of 5 mA, in a rotating sequence at 48 frames per second. Monitoring variables (HR, RR, ETCO_2_, S_P_O_2_, T) were manually recorded at the beginning of each EIT recording.

### 2.6 *Post-hoc* data analysis

Analyses of the EIT data were performed using an EIT analytical software (Ibex, Sentec AG, EIT branch, Switzerland). The raw EIT data were reconstructed into anatomically accurate functional EIT images using the chicken FE model which was built according to the modified Graz consensus reconstruction algorithm for EIT (GREIT) (10, 11).

EIT data from six to ten consecutive artefact-free breaths were analysed from the 2-min recording during each recumbency position.

After the initial EIT data analysis, it became apparent that in contrast to mammals, no impedance changes between inspiration and expiration appeared within the lung ROI. The ΔZ signals were instead located in the area ventral to the lung ROI, which corresponded to the area of the cranial air sacs and the cardiac silhouette based on CT. As adaptation of the FE model was deemed not possible, analyses of the following EIT variables were computed using only the outer contour of the initial FE model:

1. The centre of ventilation (CoV) (%), which describes the geometric focal point of overall ventilation as a percentage, from the right-to-left direction (CoV_RL_) or the ventral-to-dorsal direction (CoV_VD_). the CoV percentage represents the extension of ventilation towards the respective direction. for instance, CoV_RL_ of 0% indicates ventilation occurred predominantly in the right coelom, and 100% indicates ventilation occurred predominantly in the left region. CoV_VD_ of 0% indicates ventilation occurred predominantly in the ventral part of coelom, and 100% indicates ventilation occurred predominantly in the dorsal (9).
2. The right to left ratio (RL ratio), describes the impedance changes associated with the right (ΔZV_R_) and the left (ΔZV_L_) side of the coelom. The ratio is then calculated as ΔZV_R_/ΔZV_L_ (9).
3. Distribution of ventilation within regions (%). the global outer contour is separated into four staggered regions of area in both left and right side: dorsal (ΔZR_D_, ΔZL_D_), central-dorsal (ΔZR_CD_, ΔZL_CD_), central-ventral (ΔZR_CV_, ΔZL_CV_) and ventral (ΔZR_V_, ΔZL_V_) regions for both the left (L) and right (R) side. each region is expressed as a percentage of the total ΔZ, where the sum of all eight regions is 100% (9).
4. Tidal impedance variation (TIV) in arbitrary units (AU), a surrogate for tidal volume in mammals. It represents the global ΔZ measured between the start and end of inspiration. The TIV is therefore calculated by subtracting the impedance at the end of inspiration from the impedance at the start of inspiration (9).
5. Inspiratory time (s). This parameter is calculated by the time difference in seconds between the start and end of inspiration of the same breath.

During data collection, distinctive breathing patterns were observed when the chickens were placed into different recumbencies. In certain positions, some chickens demonstrated a temporary pause during the process of exhalation. This distinction between different patterns could be identified on the expiratory limb of the impedance curve. The temporary pause during exhalation parallels a momentary plateau in the impedance signal.

**Figure 5:**
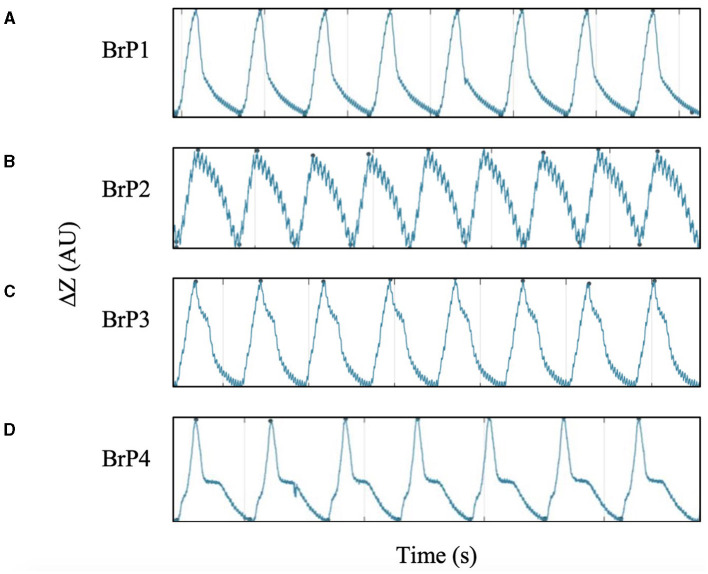
Impedance time curve for the four categories of breathing pattern (BrP), defined by the shape of the expiratory curve. All chickens demonstrated one of the four BrP uniformly throughout data collection. Minute fluttering signals can be observed superimposing on the impedance time curve. **(A)** BrP1 has a concave-linear expiratory curve without impedance pause. **(B)** BrP2 has a convex-linear expiratory curve without impedance pause. **(C)** BrP3 with an impedance pause event characterised by a distinct inflexion point at the early phase of expiration, when impedance change remained >50%. **(D)** BrP4 with an impedance pause event characterised by a distinct inflexion point at the late phase of expiration, when impedance change was ≤ 50%.

Based on these observations, breathing patterns (BrP) were classified into four categories (BrP1-4) based on the shape of the expiratory limb of the impedance curve, the presence of the pause, and the timing of the pause during expiration are detailed in Figure 5. Each chicken was only classified to have a specific BrP at each specific recumbency position when at least 95% of all analysed breaths fell into one of the four defined categories.

### 2.7 Statistics

Data were analysed with Stata (version 16.1, StataCorp). Normality of outcome variables were assessed by the Shapiro-Wilks test. The mean values of the outcome variables including CoV_RL_, CoV_VD_, RL ratio, regions of ventilation (ΔZR_D_, ΔZR_CD_, ΔZR_CV_, ΔZR_V_, ΔZL_D_, ΔZL_CD_, ΔZL_CV_, ΔZL_V_) in percentage relative to total ΔZ, TIV, the inspiratory time and respiratory rate at each recumbency were included in the statistical analyses. The outcome variables were visually inspected for outliers. None were found.

For each outcome variable, except for ΔZL_CV_, a linear mixed model with a chicken-specific random intercept was fitted to the data. Autocorrection function plots of the residuals confirmed no autoregressive pattern in the data. For ΔZL_CV_, a fixed-effect linear model was used (as a likelihood ratio test confirmed no differences to a linear mixed model) and the addition of an auto-regressive correlation structure to the residuals was not required. The four recumbency positions were included as covariates for all outcome variables. A likelihood ratio test was used to confirm that each chicken's previous recumbency position did not need to be included as a covariate. The final linear mixed model for each outcome variable used restricted maximum likelihood (REML) estimation. Quantile-normal plots were used to assess the normality of the distribution of the residuals once the models were fitted for each outcome variable. Plots comparing the standardised model residuals to the fitted values were used to visually inspect for highly influential observed values; none were found. Wald tests were used to compare recumbency positions for each parameter. No adjustments were made to the p-values. *P* ≤ 0.05 was considered statistically significant.

For the analysis of the breathing patterns, the number of chickens demonstrating each of the four categories, at each recumbency, were counted. Fisher's exact test was used to compare the number of chickens per category between recumbencies. *P* ≤ 0.05 was considered statistically significant.

## 3 Results

All fourteen recruited chickens completed their respective procedures: CT (*n* = 4) and EIT study protocol (*n* = 10). General anaesthesia for all chickens was uneventful. In one chicken, the EIT data from left lateral recumbency was excluded as no data was recorded due to artefactual signal interference. Therefore, ten sets of data were available for dorsal, ventral and right lateral recumbencies, and nine sets of data were available for left lateral recumbency. No issues were encountered in the physical application of the EIT belt. No skin damage or irritation was noted from the belt placement or the use of low-conductive ultrasound gel.

Results for all physiological variables recorded during the EIT study protocol are presented in Table 1 as medians and inter-quartile ranges.

**Table 1:** Physiologic variables (presented as medians and inter-quartile ranges) of the isoflurane-anaesthetised chickens in dorsal, ventral, right and left lateral recumbency.

	**Recumbency position**			
**Variable**	**Dorsal**	**Ventral**	**Right lateral**	**Left lateral**
HR (beats/min)	247 (229, 321)	217 (199, 263)	232.5 (221, 273)	225.5 (197, 272)
RR (breaths/min)	8 (7, 11)	10 (8, 12)	8.5 (7, 12)	8 (7, 11)
ETCO_2_ (mmHg)	53.5 (49, 63)	38 (33, 46)	36 (28, 42)	40 (34, 58)
S_p_O_2_ (%)	98 (98, 99)	99 (99, 99)	98.5 (98, 99)	99 (99, 99)
Cloacal T (°C)	39.8 (38.8, 40.1)	39.2 (39, 40.3)	39.15 (38.1, 39.6)	38.6 (37.8, 39.7)

HR, heart rate; RR, respiratory rate; ETCO_2_, end-tidal partial pressure of carbon dioxide; S_*P*_O_2_, peripheral haemoglobin saturation with oxygen; T, temperature.

### 3.1 CT analysis

**Figure 6:**
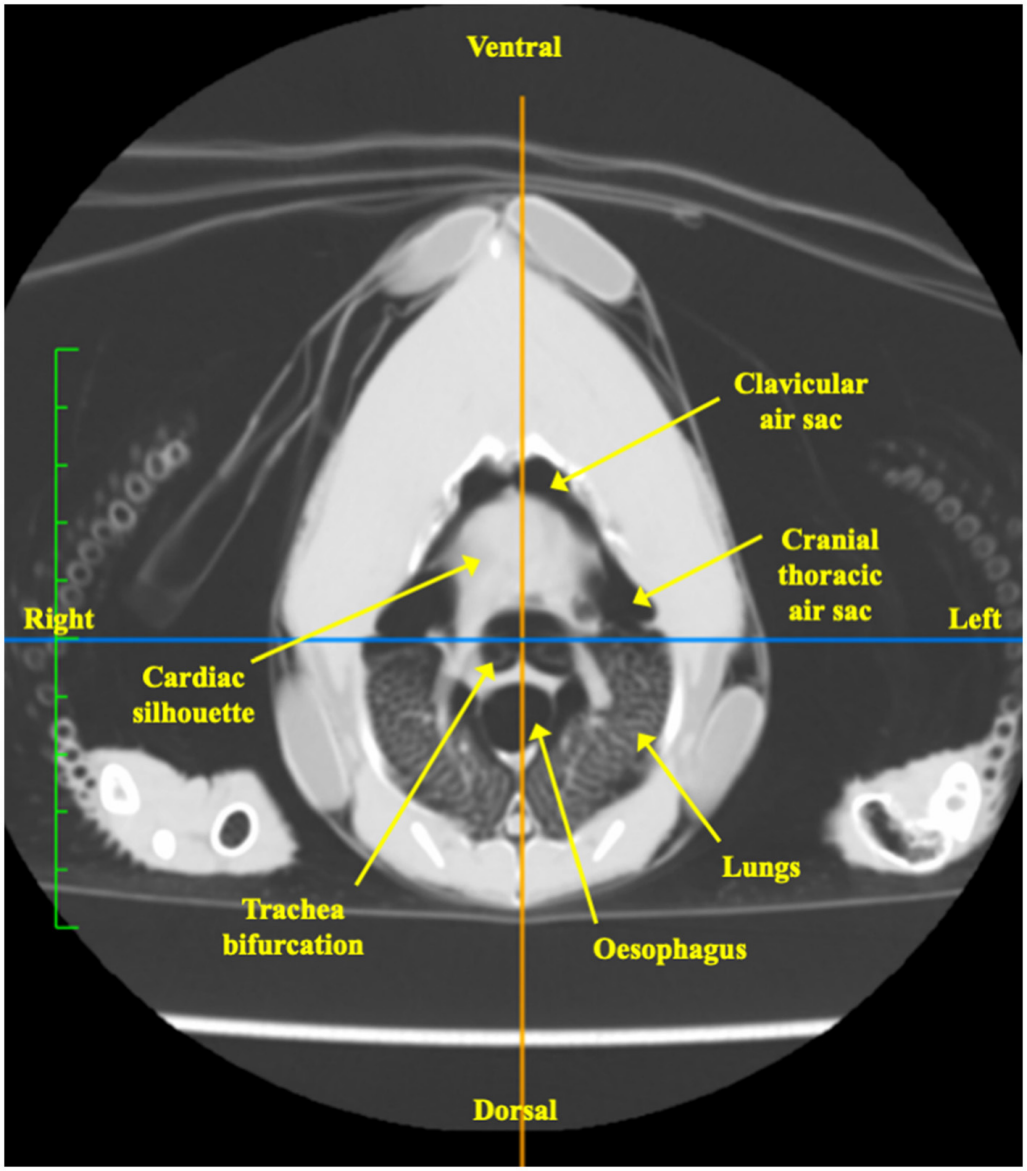
Computed tomographic image in the transverse plane of one chicken at the level of the EIT belt, immediately caudal to the shoulder joints. The organs transected at the level of the belt include the cardiac silhouette, lungs, clavicular air sac, cranial thoracic air sac, tracheal bifurcations and oesophagus.

The tomographic image slice corresponded to the level of the sham belt (Figure 6). The organs transacted at this level were visually identified and included the cardiac silhouette, the lungs, the cranial air sacs group which itself comprised of the clavicular air sac and the bilateral paired cranial thoracic air sac, tracheal bifurcation and the oesophagus (32).

During visual comparison of the inspiratory and expiratory phases, the body contour and skeletal structures remained static in position and size (Figures 3A, B). The mean area of the lungs was 7.7% larger during inspiration than in expiration, as measured by an online area calculator (SketchAndCal, Icalc Inc). The area of the air sacs was also increased at inspiration when compared to expiration. The area of the cardiac silhouette was decreased at inspiration when compared to expiration. This observation was consistent in all four chickens.

### 3.2 EIT signal analysis

The application of EIT for real-time ventilation monitoring in chickens was possible when using the purpose-designed electrode belt and EIT software. On visual inspection, the impedance change over time plotted by the EIT software was synchronous with inspiration and the manually recorded respiratory rate. It was therefore determined that the recorded ΔZ was due to ventilation and represented the TIV. These cyclical changes in ΔZ were observed for all breaths in all chickens. Small fluttering signals were observed superimposing on the impedance-time curve in all chickens (Figure 3). The frequency of these signals was synchronous to the heart rate and represented cardiac-related signals. These cardiac-related signals were minuscule in magnitude and did not affect recognition of the TIV by the EIT software during analysis.

The EIT data were successfully reconstructed into anatomically accurate functional tomographic images using the outer contour of the chicken-specific FE model. On these functional images, no ΔZ signal was observed in the lung ROI throughout the respiratory cycle. Based on comparison between the EIT images using the FE model and the corresponding CT images, the EIT signals situated in the area ventral to the lung ROI represented ΔZ in the area of the cranial air sacs and the cardiac silhouette instead (Figure 2E). As the cardiac-related signals were only minimal, the ΔZ recorded predominantly represented the TIV of the cranial air sacs.

### 3.3 Distribution of ventilation

The use of EIT in chickens demonstrated good responsiveness to recumbency changes, detecting significant changes in the distribution of ventilation. All results and significant changes between the four recumbencies are presented in Table 2.

**Table 2:** Electrical impedance tomography variables (mean and 95% confidence interval) of the coelom in isoflurane-anaesthetised chickens in dorsal, ventral, right, and left lateral recumbencies.

	**Recumbency position**			
**Variable**	**Dorsal**	**Ventral**	**Right lateral**	**Left lateral**
CoV_RL_ (%)	48.8 (47.6–50.1)^a, b^	48.4 (47.2–49.7)^c^	51.9 (50.7–53.1)^a, c, d^	46.9 (45.6–48.2)^b, d^
CoV_VD_ (%)	52.3 (51.1–53.5)^a, b^	55.0 (53.8–56.2)^a, c^	53.1 (51.9–54.3)^c^	54.2 (53.0–55.5)^b^
R:L ratio	1.20 (0.96–1.44)^a, b^	1.27 (1.03–1.51)^c, d^	0.83 (0.59–1.07)^a, c, e^	1.61 (1.36–1.86)^b, d, e^
ΔZR_V_ (%)	0.31 (0.20–0.42)^a^	0.18 (0.08–0.29)^a, b^	0.35 (0.24–0.45)^b^	0.31 (0.20–0.42)
ΔZR_CV_ (%)	21.3 (16.4–26.2)^a, b^	16.1 (11.2–21.0)^a, c^	16.5 (11.6–21.4)^b, d^	22.5 (17.5–27.4)^c, d^
ΔZR_CD_ (%)	31.3 (27.5–35.0)^a, b^	37.5 (33.7–41.2)^a, c^	24.2 (20.5–27.9)^b, c, d^	36.0 (32.1–39.9)^d^
ΔZR_D_ (%)	1.03 (0.37–1.68)^a^	1.26 (0.60–1.91)^b^	2.18 (1.52–2.84)^a, b^	1.85 (1.16–2.54)
ΔZL_V_ (%)	0.79 (0.5–1.08)^a, b^	0.31 (0.02–0.60)^a, c, d^	0.62 (0.33–0.91)^c^	0.53 (0.24–0.83)^b, d^
ΔZL_CV_ (%)	24.4 (20.9–27.9)^a, b^	19.6 (16.1–23.1)^c^	30.1 (26.6–33.6)^a, c, d^	16.5 (12.8–20.1)^b, d^
ΔZL_CD_ (%)	19.6 (16.9–22.2)^a, b^	23.9 (21.3–26.6)^a, c^	23.5 (20.8–26.2)^b, d^	20.0 (17.3–22.8)^c, d^
ΔZL_D_ (%)	1.33 (0.72–1.94)^a, b^	1.15 (0.54–1.76)^c, d^	2.53 (1.92–3.14)^a, c^	2.24 (1.60–2.88)^b, d^
TIV (AU)	6.65 (5.30–8.00)	5.62 (4.27–6.96)	6.38 (5.03–7.72)	6.37 (4.98–7.76)
Inspiratory time (s)	2.31 (1.82–2.80)^a^	2.57 (2.08–3.05)	2.70 (2.21–3.19)^a^	2.50 (2.00–2.99)

EIT, electrical impedance tomography; CoV_RL_ and CoV_VD_, centre of ventilation right to left and ventral to dorsal, respectively; R,L ratio, right to left ratio; ΔZR and ΔZL, impedance change within the right and left regions, respectively, in percentage of total impedance change; ΔZR_D_, ΔZR_CD_, ΔZR_CV_, ΔZR_V_, ΔZL_D_, ΔZL_CD_, ΔZL_CV_, ΔZL_V_, impedance change within right and left (dorsal, central-dorsal, central-ventral, ventral) regions, respectively, in percentage of total impedance change; TIV, tidal impedance variation; AU, arbitrary units. Means sharing the same superscript (^a, b, c, d, e^) within a row indicates statistically significant differences (P < 0.05).

### 3.4 Tidal impedance variation, respiratory rate and inspiratory time

No significant difference was found in TIV and RR across all four recumbencies (Tables 1, 2). No significant difference was found in the inspiratory time between dorsal and ventral recumbencies, nor between right and left lateral recumbencies. Inspiratory time in dorsal recumbency was significantly higher than in right lateral recumbency (*P* = 0.006).

### 3.5 Respiratory pattern

**Figure 7:**
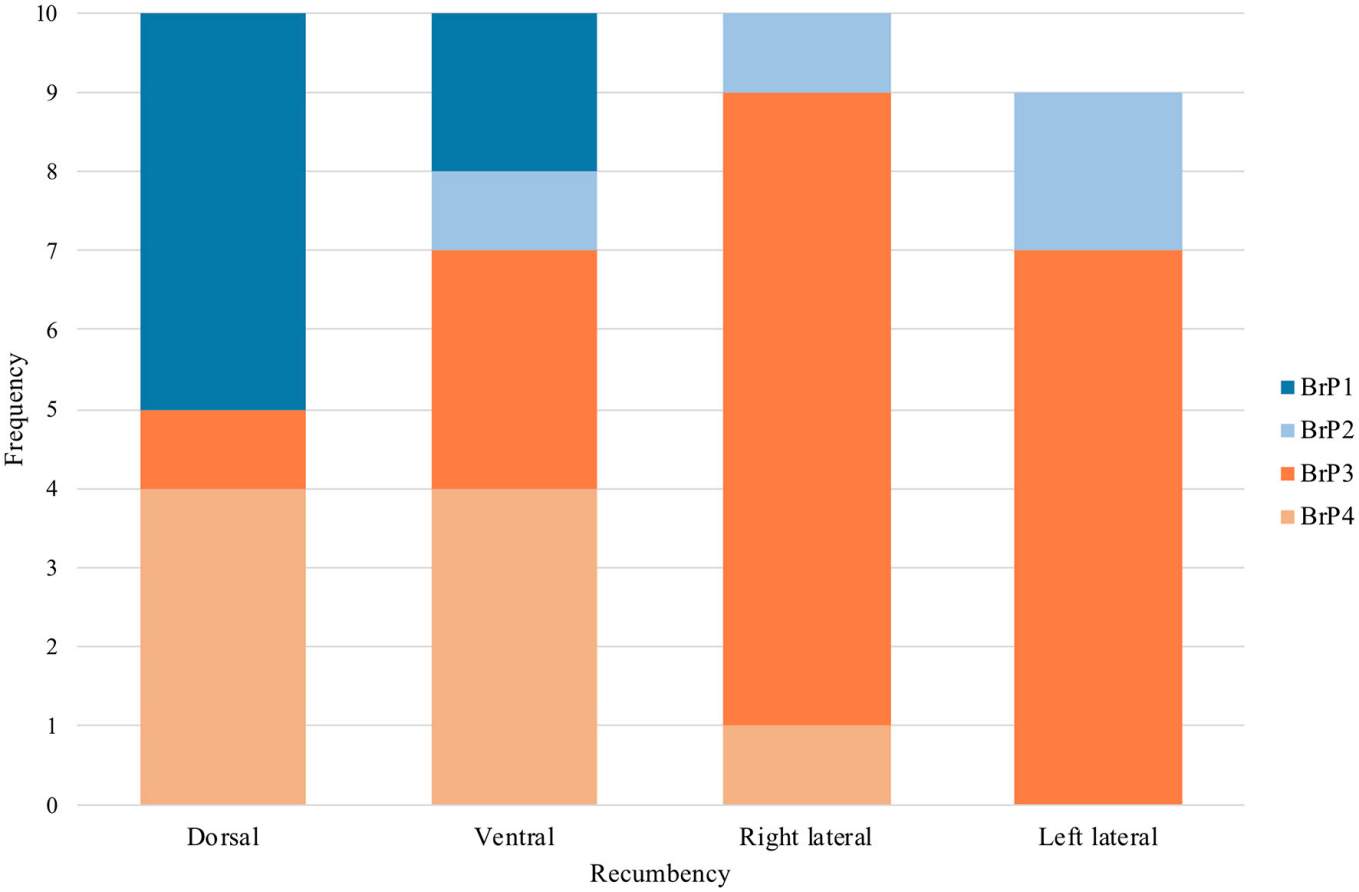
The frequency of subjects that demonstrated each of the four categories of breathing pattern (BrP), as defined by the shape of the expiratory impedance time curve, in dorsal, ventral, right and left lateral recumbencies, in the ten isoflurane-anaesthetised chickens enrolled in the study protocol. One chicken in left lateral recumbency was excluded due to missing data from artefactual signal interference.

All chickens displayed only one of the four BrP categories (Figure 5) throughout the 2 min of data collection while in a particular position of recumbency. The number of chickens with a particular BrP at each recumbency was significantly different to the frequency expected (*P* = 0.001) (Figure 7).

In dorsal recumbency, the majority of the chickens had either BrP1 (5/10) or BrP4 (4/10), with one chicken displaying BrP3. In ventral recumbency, most chickens had either BrP4 (4/10) or BrP3 (3/10), with two chickens displaying BrP1 and one chicken had BrP2.

In both lateral recumbencies, most chickens had BrP3 (15/19). In right lateral recumbency, one chicken each displayed BrP2 and BrP4, while the rest had BrP3 (7/9). In left lateral recumbency, two chickens had BrP2, with the rest displaying BrP3 (8/10).

## 4 Discussion

### 4.1 Summary of findings

This study has demonstrated that the technology of EIT is applicable in chickens to monitor ventilation of the cranial air sacs. Significant changes in the EIT variables of distribution of ventilation (CoV_RL_, CoV_VD_, RL ratio, and regional ΔZ's) were detected when the position was changed. Significant changes in respiratory pattern were noted when chickens were moved into different recumbency positions. However, no significant differences were found in TIV and RR between the recumbency positions.

### 4.2 Feasibility and practical considerations

This is the first study to trial and demonstrate the application of EIT in birds. The practical decisions elected for the study design was based on previous experiences in mammals. In the past, EIT data was obtained with the use of individual electrode pads in neonates and dogs (33, 34), this has been superseded with the use of an electrode belt in veterinarian medicine as the conventional approach that conveniently ensures uniform placement of electrodes across study subjects (9). In this study, the belt placement was visually inspected at all times to ensure no belt-induced deformation of the body that may interfere with ventilation. The use of a textile EIT belt has also been shown to provide suitable and long-term use without causing discomfort and distress in preterm infants (35).

The EIT belt utilised was fitted with gold-plated pin arrays (31). Gold electrodes has been shown to provide the least skin contact impedance due to its high electrical conductivity when compared to zinc-based electrodes (36). Pin arrays electrodes were ideal for chickens to accommodate their relatively small thoracic circumference. While skin damage from pinned or spiked electrodes is a theoretical concern, this was not observed in this study, and was not reported in previous studies in dogs and steers (33, 36). However, the weight of the animal and recumbency factors may contribute to the potential risk of skin damage. The feasibility of other electrode types in birds should be investigated in future studies.

The optimisation of EIT signals was accomplished by multiple strategies. Firstly, the feathers were depilated to maximise the electrode-skin contact. Similarly, fur clipping has been documented in previous canine studies (33). Secondly, the application of low-conductive ultrasound gel also aids the electrode-skin contact by abolishing air pockets between the electrode and the skin. Importantly, it also encourages the current to penetrate the skin and the thorax, and prevents bypass current from conducting on the skin surface between electrodes, which may otherwise occur with high conducting gel (9). Thirdly, stay sutures were used to maintain belt positioning and to prevent movement-related interference. This is however not a routine practise in other veterinary species. Further studies should evaluate the feasibility of EIT in birds without the requirement to place stay sutures and feather depilation, to improve its accessibility for client-owned birds. Ultimately, good data signals were collected in all but one of the chickens while in left lateral recumbency. A previous equine study reported electrical artefacts secondary to the use of electrocautery, but this was not utilised in this study (19). The exact cause of the signal interference in this case was unknown.

### 4.3 Origin of impedance signals

The impedance signals that originated from the air sacs were distinctly observed in the chickens in this study. There is contention about the origin of impedance signals. In mammals, it has been argued that ΔZ could be a result of the stretch and deformation of tissue and blood secondary to alveolar expansion, instead of an increase in gas volume (25, 26). Yet, the detection by EIT of well-defined ΔZ within the thin and poorly vascularised air sacs was shown to be possible in this study (27). This supports that the ΔZ signal could be generated from a change of gas volume rather than being solely derived from the stretching of tissue and blood (37).

### 4.4 Impedance signals in the avian model

The tomographic image of the thorax was essential in identifying the anatomical structures transected at the level of the EIT belt. The image showed that both the unpaired clavicular and paired cranial thoracic air sacs were clearly included. In contrast, the involvement of the cervical air sac was unlikely to be included in the image as the bulk of the cervical air sac lies cranial to the third thoracic vertebra (32). Regardless, the cervical air sac contributes minimally to ventilation and its exclusion should not affect the analysis of ventilation of the cranial air sacs (6).

The contours of the lung ROI were clearly defined on the tomographic image without any major organs overlapping, allowing segmentation to be done with precision using the dedicated software (28). No ΔZ was detected within the lung's ROI. The lack of signal is consistent with avian respiratory physiology where gas exchange and ventilation are functionally separated. During both inspiration and expiration, gas flows are driven through the lungs in a unidirectional manner by the compliant air sacs. The lungs, where gas exchange occurs, are considered non-compliant and would therefore remain isovolumetric (6, 38). As there is no difference in gas volume within the parabronchi between the onset of inspiration and end-inspiration, it follows that no ΔZ would be detected. In this study, the area of the lungs was noted to be larger during inspiration than in expiration on CT images. While this is inconsistent with the consensus that the lungs remain static in size, it is in agreement with a previous study in ducks that used tantalum markers and radiographs to show that lung thickness cyclically fluctuated during ventilation (39). Additionally, the movement of the viscera within the coelom may have contributed to this discrepancy in lung area between inspiration and expiration, even though the belt was tethered to the external chest wall. A similar phenomenon has been reported in dogs where the movement of organs within the thorax and abdomen under the EIT belt may have contributed to variations in the EIT variables (14).

In contrast to the lungs, segmentation of the air sacs as an independent ROI for the FE model was not achievable. The dynamic superimposition of the cardiac silhouette and air sacs areas between inspiration and expiration made independent segregation of these two structures impossible. It should be noted that while the transverse CT image represents a two-dimensional slice, the EIT image actually represents a three-dimensional lens-shaped slice at the level of the belt, with increasing thickness in the central region of the slice (Figure 1). Isolation and removal of the cardiac silhouette from the area of interest would exclude a considerable proportion of the impedance signals originating from the air sacs.

On the CT images, the cardiac silhouette was pushed caudally during inspiration by the inflation of cranial air sacs, towards the caudal limits of the lens-shaped slice. This finding was consistently demonstrated by the transverse end-inspiratory CT slice in which the area of the cardiac silhouette was smaller (Figure 3). Conveniently, the cranial air sacs were then located at the thickest portion of the EIT slice during inspiration. The quality and amplitude of TIV were therefore maximised, with minimal contribution of the cardiac related signals towards the overall recorded ΔZ. Based on this observation, combining the cranial air sacs and the cardiac silhouette together as one single ROI may be a possible option. A ROI that is specific to the cranial air sacs would allow a more localised assessment of the distribution of ventilation within specific regions of the cranial air sacs. The utilisation of the global contour as performed in this study will inadvertently include areas with unrelated impedance signals, and may decrease specificity of the changes in the distribution of ventilation (9).

### 4.5 Distribution of ventilation

The results of this study confirmed the first hypothesis that EIT can detect subtle ventilatory changes between different recumbency positions. The results also confirmed the second hypothesis that recumbency significantly affects EIT variables. Concurrently, insights are also provided into the effects of recumbency change on ventilation and its distribution. A consistent shift in ventilation towards the non-dependent area was noted. In dorsal recumbency, the CoV_VD_, ΔZR_CD_ and ΔZL_CD_ were significantly less than that in ventral recumbency. In lateral recumbencies, ventilation tended to shift towards the non-dependent central regions, as indicated by the significantly lower ΔZR_CD_ and ΔZR_CV_ in the right lateral than in the left lateral recumbency, and significantly higher ΔZL_CD_ and ΔZL_CV_ in the right lateral than in the left lateral recumbency (Table 2). Similar phenomena have been reported in mammalian species, including dogs (14) and rhinoceroses (40) during spontaneous ventilation. While mammals do not have air sacs, each mammalian alveolus is conceptually comparable, but functionally distinct to the air sacs as both represent the compliant structures that expand and shrink during the ventilatory cycle. These shifts in ventilation can be explained by the changes in compliance of the air sacs, attributed to factors such as a lack of diaphragm, visceral organ compressions, alteration of the body wall shape and respiratory muscle relaxation influencing the functional residual capacity within the air sacs.

In mammals, the dynamic changes in pulmonary compliance are best explained by a pressure-volume curve, which plots the volume in the pulmonary system at specific airway pressure points. The curve is typically sigmoidal in shape: at normal expanding pressure of −5 to −10 cmH_2_O pressure during spontaneous ventilation, the gradient of the curve is steep, indicating good compliance; at the extremes of low and high pressure, the gradient of the curve is flat indicating low compliance (41). It is likely that in the recumbent position, the dependent portion of the air sacs shift from the steep compliant part of the pressure-volume inflation curve to the lower flat non-compliant part of the curve, thereby allowing the non-dependent portion of the air sacs to be preferentially filled. However, in one canine EIT study, recumbency affected the distribution of ventilation only when dogs were anaesthetised, not when they were conscious (14). Our study supports that general anaesthesia, perhaps by way of muscle relaxation, alters the distribution of ventilation in birds comparable to the way observed in mammals.

### 4.6 Tidal impedance variation

The parameter TIV (AU) has been used as a surrogate for tidal volume based on their linear relationship that has been observed in multiple domestic species (17–20). Similarly, TIV should also represent the volume of the cranial air sacs in birds, as captured by the lens shaped EIT in this study. The results in this study demonstrated no difference in the TIV of the cranial air sacs between the four recumbencies. This indicated that the total gas volume in the cranial air sacs was not influenced by a change in recumbency, despite the differences in the distribution of ventilation as described above.

Alterations in the total air sacs volume and tidal volume secondary to changes in recumbency have been documented (42–44). Up to 40%−50% decreases in tidal volume have been reported in chickens when changed from standing to dorsal recumbency (42), postulated to be due to compression of the air sacs by visceral organs. This has led to clinical recommendations to avoid dorsal recumbency in birds (1, 6). On the contrary, one study refuted this notion as a significantly greater tidal volume and greater P_a_O_2_ was found in dorsal recumbency than in right lateral recumbency, in anaesthetised red-tailed hawks (44). Other authors also suggest the avoidance of ventral recumbency to minimise restriction of the keel movement (42, 43, 45, 46). In the present study, only the cranial air sacs were investigated. Previous studies have implied that the caudal air sacs are preferentially affected during recumbency changes, due to the presence of viscera such as the reproductive tract in the caudal coelom (6, 42). However, the impact of caudal air sacs compression on the total tidal volume may vary among different bird species, as the relative capacity of individual air sacs varies widely between species (22). Regardless, specific changes to the caudal air sacs were not investigated in the present study. Further studies are required to verify the effect of recumbency on caudal air sacs ventilation, and the total tidal ventilation. The additional use of spirometry would also be useful to measure tidal volume and compare it to cranial and caudal air sacs ventilation.

### 4.7 Respiratory pattern

The use of EIT allowed objective quantification of the changes in respiratory pattern. In this study, the method of categorising BrP using the morphology of the expiratory impedance curve was based on that reported in rhinoceros and horse EIT studies (40, 47). It was interesting to note that in both right and left lateral recumbencies, the majority of the chickens demonstrated early expiratory breath holding (BrP3). In comparison, a wider variety of BrP was seen when the chickens were in dorsal or ventral recumbencies. The mechanisms that explains why these respiratory patterns change is unclear. Alterations to the respiratory patterns were previously suggested to be a compensatory mechanism to auto-recruit collapsed pulmonary tissues in horses and rhinoceroses (40, 48). A similar compensatory mechanism is possible in birds to preserve the ability of the air sacs to maintain appropriate flow through the parabronchi during both inspiration and expiration.

Alternatively, it is possible that these changes to the respiratory patterns were direct consequences of positioning. Inspiration and expiration are active processes in birds and so the action of the respiratory muscles involved during expiration may be compromised when the chickens are in recumbencies other than the upright position. The compression of the dependent coelom wall, and changes in air sacs compliance can affect flow patterns through the parabronchi by increasing the resistance to flow (49–51). Additionally, the expiratory flow by the cranial air sacs have been theorised to make important contribution to the functional valving that creates unidirectional flow, along with other factors such as gas flow variables and the structural geometry between the air sacs and airways (52). The alteration to the expiratory flow is therefore logical when recumbency is changed, as the ability to generate the prototypical flow is compromised.

### 4.8 Limitations and future studies

A limitation of this study was the inability to produce a ROI specific for the cranial air sacs, and so data analysis was performed using only the global outer contour instead. The distribution of ventilation within specific regions may therefore be occupied and influenced by non-respiratory related areas of the coelom. Development of a cranial air sacs ROI would provide more precise information on the distribution of ventilation. As previously mentioned, development of a ROI incorporating both cranial air sacs and the cardiac silhouette may be a feasible solution, as the cardiac-related signals contributed minimally to the global ΔZ.

Another limitation of this study is that only a homogenous population of chickens was recruited. Inclusion of chickens of other breeds, age groups and sex would permit further investigation into the utility of EIT in chickens. Birds show immense biodiversity in their form and function. The feasibility of applying EIT to other birds is therefore currently unknown.

Only the cranial air sacs were investigated in this study. Future studies should investigate the use of EIT on the caudal air sacs. Techniques such as the use of two-plane EIT should be considered for simultaneous detection of the caudal air sacs ventilation. The use of two-plane EIT has been described in horses, which allows more tissues to be captured in the longitudinal axis, as opposed to a single transverse slice with standard one-plane EIT as used in this study. The impedance signals obtained by the two-plane EIT can then be reconstructed into three-dimensional images of the thorax (53). Measurement of both the cranial and caudal air sacs will be important to allow a global assessment of the entire avian ventilatory system. For instance, the cranial and caudal air sacs groups have been shown to contribute equally to tidal ventilation in anaesthetised ducks (46).

The effects of recumbency on ventilation with EIT variables were investigated as a secondary outcome. However, the impact that recumbency had on gas exchange was beyond the scope of the study. Physiological variables, such as ETCO_2_ was primarily recorded for the standard monitoring during anaesthesia. In this study, a side stream capnograph and a Mapleson D, non-rebreathing system was used for the EIT study protocol. As the capnograph adapter was placed adjacent to the fresh gas outflow, the high oxygen flow required to prevent rebreathing can induce turbulence and dilution of capnography reading, rendering ETCO_2_ not representative of P_a_CO_2_ (54, 55). The agreement between ETCO_2_ and PaCO_2_ has been shown to be unreliable in ventilated anaesthetised birds with a non-rebreathing system (8). It was therefore decided to not statistically explore ETCO_2_ to avoid misleading interpretations of the effect that recumbency has on ventilation.

The optimal position of recumbency for the respiratory system during avian anaesthesia is yet to be determined. In clinical practise, the recumbency assigned depends mainly on the nature of the procedure to optimise surgical access, and to a lesser extent the impact on the cardiorespiratory system. In future studies, assessment of gas exchange by arterial blood gas analyses; assessment of the cardiovascular system by measuring cardiac output; and concurrent assessment of the caudal air sacs, should be considered to determine the optimal recumbency position in birds under general anaesthesia.

Cardiovascular monitoring using EIT should also be explored. Measurement of pulse rate was possible using cardiac-related signals in horses (56). Furthermore, haemodynamic signals from the aorta have been shown to provide information on preload and fluid responsiveness in anaesthetised pigs (57). These functions may also be possible in chickens, as clear cardiac-related signals were incidentally noted on the impedance-time curve in this study. Further research is required to develop algorithms that filter and analyse these cardiac-related signals.

The practical application of EIT for clinical respiratory monitoring in birds should also be considered. The unique ability of EIT to assess ventilation in real-time is advantageous in avian clinical practise, as monitoring is often limited. Future topics of investigations include the development of a more ergonomic and robust belt that accommodates various sizes of birds, methods to improve electrode contact and signal detection that does not require the use of stay sutures and feather plucking. More bird species-specific FE models will need to be developed.

## 5 Conclusion

EIT can be used in chickens to monitor ventilation by detecting impedance signals that are synchronous to ventilation. It can also be used to detect changes in EIT variables when the position of recumbency is changed. EIT can provide valuable information about the distribution of ventilation and also a surrogate measure for the tidal volume in the cranial air sacs. Recumbency affected the distribution of ventilation, with the tendency to shift towards the non-dependent air sacs. Recumbency significantly influenced BrP, but did not affect cranial air sacs TIV and respiratory rate. Further studies are required to investigate its utility for clinical practise.

## Data availability statement

The original contributions presented in the study are included in the article/supplementary material, further inquiries can be directed to the corresponding author.

## Ethics statement

The animal study was approved by Animal Ethics Committee, Murdoch University. The study was conducted in accordance with the local legislation and institutional requirements.

## Author contributions

MM, HL, and GM designed the research and performed experiments. AMW, HL, ADW, RB, TH, and MM analysed the data. RB and TH did the statistical analysis. AMW and MM drafted the manuscript. All authors interpreted the results, edited, revised, and approved final version of the manuscript.
